# ENCoM server: exploring protein conformational space and the effect of mutations on protein function and stability

**DOI:** 10.1093/nar/gkv343

**Published:** 2015-04-16

**Authors:** Vincent Frappier, Matthieu Chartier, Rafael J. Najmanovich

**Affiliations:** Department of Biochemistry, Faculty of Medicine and Health Sciences, University of Sherbrooke, Sherbrooke, Quebec, J1H 5N4, Canada

## Abstract

ENCoM is a coarse-grained normal mode analysis method recently introduced that unlike previous such methods is unique in that it accounts for the nature of amino acids. The inclusion of this layer of information was shown to improve conformational space sampling and apply for the first time a coarse-grained normal mode analysis method to predict the effect of single point mutations on protein dynamics and thermostability resulting from vibrational entropy changes. Here we present a web server that allows non-technical users to have access to ENCoM calculations to predict the effect of mutations on thermostability and dynamics as well as to generate geometrically realistic conformational ensembles. The server is accessible at: http://bcb.med.usherbrooke.ca/encom.

## INTRODUCTION

Proteins are dynamic objects with movements ranging from sub-rotameric side-chain movements to domain movements intrinsically associated to their function. Among the main computational techniques to study protein dynamics are molecular dynamics (MD) and normal mode analysis (NMA). The following properties are common to both techniques: (i) both can be used to explore the conformational space; (ii) may use the same force fields and the accuracy of the simulation depends on the quality of the potential; (iii) both techniques are as exact descriptions of the dynamics as the level of detail of the representation of the protein structure and the force field used permits. The major difference between MD and NMA is that the former produces an actual trajectory in conformational space while the later produces a basis set of movements described as a set of normal modes (Eigenvectors) and associated frequencies (Eigenvalues) with which individual points in conformational space can be sampled. Every possible conformational change of a protein from the starting equilibrium structure can be described as a linear combination of all Eigenvectors modulated by specific amplitudes. Therefore, NMA produces the set of possible movements whereas MD provides an actual trajectory. The modes associated to the slowest frequencies are the most energetically accessible.

Coarse-grained NMA methods use reduced amino acids representations, for example, one point mass per amino acid. Different levels of simplification in the representation of protein structures exist. However, with the exception of ENCoM (1) and VAMM (2), coarse-grained NMA models do not account for the nature of amino acids and are therefore sequence agnostic (3–5). For example, in the widely used Anisotropic Network Model (ANM) (4), all residues within a given distance threshold (usually 18 Å), are connected by springs with equal spring constants independent of the nature of the amino acids involved or even if they are actually interacting or not.

Our group recently introduced ENCoM, a coarse-grained NMA method that accounts for the nature of amino-acids through the inclusion of a pairwise atom-type term proportional to the surface area in contact between heavy-atoms in the potential. The more realistic representation of intramolecular interactions in ENCoM results in more accurate predictions of conformational changes in terms of calculated squared overlap (the extent that movement in a given normal mode direction drives the structure from a starting state toward a target one). Specifically, compared to ANM using the PSCDB (6), a database for protein structural change upon ligand binding, we obtain an average increase in squared overlap of 28% on 117 coupled domain movements and 60% on 236 cases of coupled loop movements (1).

As the first coarse-grained NMA method to account for the type and extent of pairwise atomic interactions, ENCoM can be used to calculate vibrational entropy differences as a result of mutations (1). ENCoM was compared (1) to several existing methods, notably FoldX3.0 (7), Rosetta (8), DMutant (9) and PoPMusic (10), Eris (11), CUPSAT (12), I-Mutant (13) and AUTO-MUTE (14) on a data set of 303 manually curated mutations (10). Although not the best overall predictive method when considering both stabilizing and destabilizing mutations together, ENCoM proved to be the most self-consistent and least biased method. ENCoM and DMutant had the best performance on the subset of 45 stabilizing mutations compared to other methods whose predictions in this case were as good or worse than a random model. Classic coarse-grained NMA models predicted every mutation as neutral since by definition two identical structures irrespective of their sequences will generate identical sets of Eigenvectors and Eigenvalues. Lastly, ENCoM can predict local variations in dynamics. As a proof of concept, ENCoM was used to predict the effect of the G121V mutation in dihydrofolate reductase (DHFR) from *E. Coli*. Although this mutation has only a modest effect on protein stability and is 15 Å away from the binding site, it disrupts catalytic activity 200 fold through allosteric effects. ENCoM calculations are consistent with experimentally observed variations in NMR S^2^ values (15) for the effect of this mutation with a Pearson correlation of 0.6 (1). More recently, ENCoM was used to compare a large data set of structurally identical ortholog mesophile/thermophile protein pairs to show that thermophile proteins are on average more rigid than their mesophile counterparts and that it is possible to use vibrational entropy differences to guide the selection of mutations observed between such proteins with potential uses in protein engineering (16).

**Figure 1.**
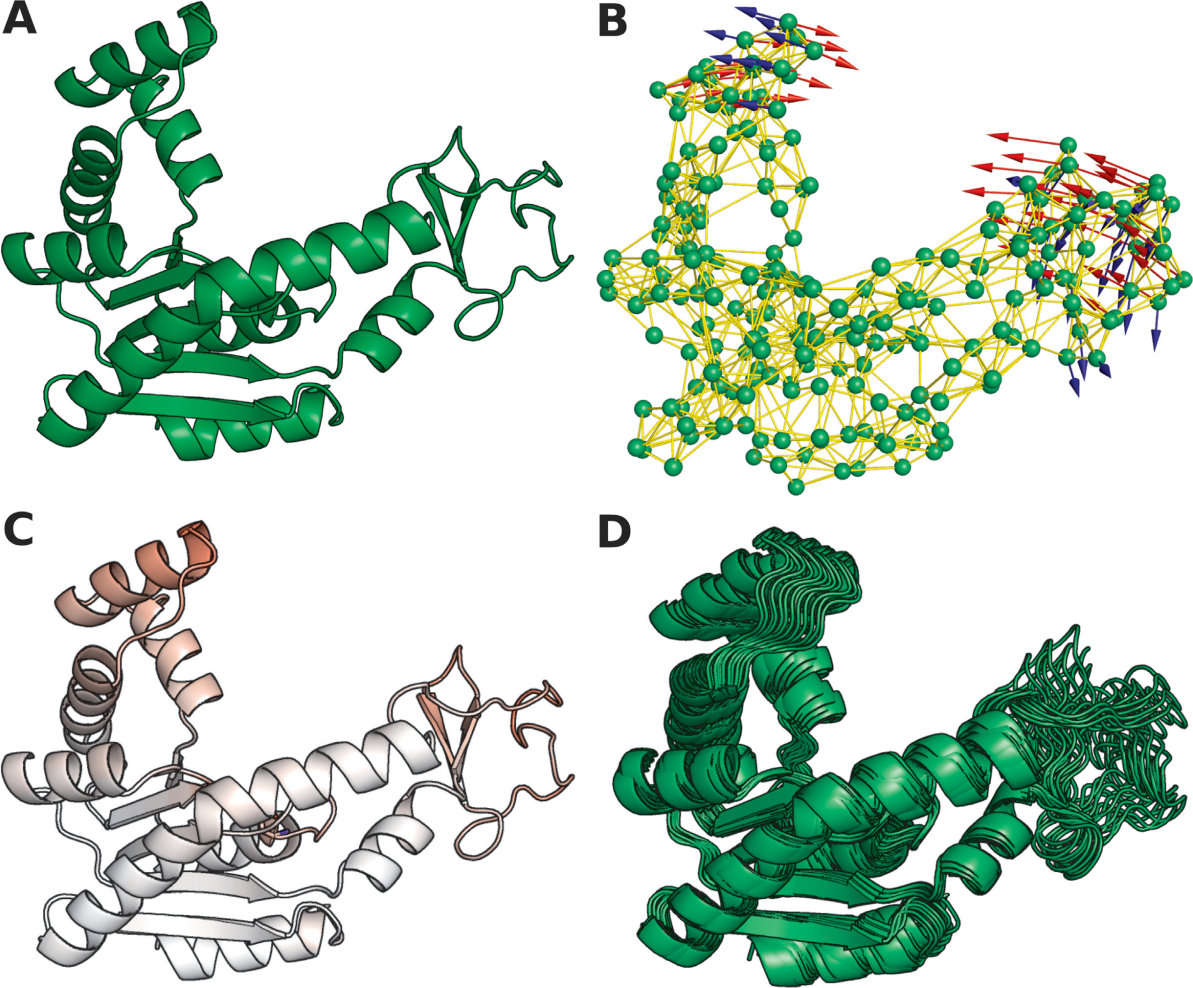
Example of using the ENCoM web-server to analyze the unbound form of adenylate kinase (PDB ID .4AKE, Panel **A**). (**B**) The Protein structure is represented as an elastic network model, where amino acids are represented by masses (green spheres) and interactions by springs (yellow stick). (**C**) The mutation K13Q causes an increase in flexibility in the regions marked in red. (**D**) The eigenvectors representing the two slowest non-trivial modes (shown in red and blue in panel B) were used to generate 49 conformations.

The level of technical skill required to use NMA methods is a barrier to their adoption even when using freely available packages such as ENCoM. A number of existing web-servers make NMA methods more accessible. These are NOMAD-ref (17), Elnemo (18), webnm@ (19), AD-ENM (20), Promode Elastic (21) ANM 2.0 (22) and iMODS (23). However, with the exception of iMODS that performs NMA in dihedral angle space, existing servers are based on Cartesian space NMA and are of limited use. In particular, some methods do not generate actual structures and those that do, provide structures that do not respect the most basic geometric constraints of bond angles and distances. Furthermore, many servers provide structures derived from single normal modes. Thus at the very least, the structures provided by existing servers require further regularization to represent geometrically plausible structures. Lastly, as a consequence of the limitations of the sequence-agnostic coarse-grained NMA models on which they are all based, none of the existing servers can be used to predict the effect of mutations on dynamics or stability. Here we present a simple, yet powerful, web-server that given a protein structure (Figure 1A) uses ENCoM to calculate coarse-grained normal modes (Figure 1B) to predict the effect of mutations on protein stability and dynamics (Figure 1C) and generate realistic, geometrically correct conformational ensembles based on the uniform exhaustive sampling of accessible modes (Figure 1D).

## IMPLEMENTATION

The ENCoM server interface is split into two input forms, depending on what type of analysis the user wants to perform: prediction of the effect of mutations or conformational sampling. In either case, users can import their own structures in PDB format or use existing PDB accession codes. Every structure analyzed with ENCoM in the server implementation is stripped of heteroatoms and hydrogen atoms and the user can choose all chains or any subset thereof. ENCoM takes in consideration inter-chain contacts for all selected chains. When using PDB accession codes, it is important to note that the ENCoM server uses the asymmetric unit, thus it is important to specify the correct chains to be included in the calculations or alternatively download the biological unit independently from a PDB repository and use this as input. Upon the submission of a job the user receives a link to a results page that is maintained for 7 days where a log shows the level of completion of the job whilst the job is running and the results once finished. If the user provides an email, a notification email is sent when the job starts and once the calculations are completed. Run time may vary from minutes to several hours and is estimated by comparing the input parameters (size of proteins and number of mutations or conformations to be evaluated) to the time taken by jobs with similar parameters. Thus, as the ENCoM server usage increases, running time estimates will improve.

### Effect of mutations on thermal stability

The mutation interface is built to allow high-throughput analysis of single-point mutations with the implementation of a simple command line interface. The user inputs the residue number or range of residues to mutate, the chain and the type of mutations. Whereas a large number of mutations may be specified at once in the input, the ENCoM server is restricted to the analysis of single mutations and models each mutation in turn as a single-point mutation. The ENCoM server utilizes the same methodology employed in our recent papers (1,16), namely we use the Modeller (24) ‘mutated’ function to model each mutant and ENCoM to predict the ΔΔG with respect to the starting structure. Particular mutations that cannot be successfully modeled are skipped. As some mutations may severely disrupt protein structure, it is necessary to keep in mind that the quality of the resulting models directly affects the calculations. While minor backbone or side-chain rearrangements are unlikely to have major effects on the analysis, if a Modeller-generated structure is entirely wrong, for example modeling a mutant that in reality cannot generate a stable fold, the results will clearly be meaningless.

As stabilizing mutations are more rare, the possibility to input a large number of mutations at once (even all 19 possibilities in each position) in the ENCoM server web interface simplifies the search for such mutations that are particularly relevant in the context of protein design. With the exception of PoPMusic, all other web-servers dedicated to the prediction of the effect of mutations on stability require that users input every position to be mutated (and the nature of the corresponding residue in the wild type) manually, making the task of exploring the effect of mutations unnecessarily time consuming.

In our previous work (1), we tested linear combinations of ENCoM with other methods but did not test combination that did not involve ENCoM. Here, we perform all possible method combinations between ENCoM, Eris, FoldX, Rosetta and DMutant as these were shown in our previous work to be the best performing, less biased methods on both stabilizing and destabilizing mutations. Some methods were excluded due to significant number of errors (CUPSAT and AUTO-MUTE), strong biases toward predicting mutation as destabilizing (I-Mutant and PoPMusic) and that had no predictive power (ANM and STeM). In order to determine which combination of methods is best, the 303 mutations from the Dehouck *et al*. data set (10) were used to create 10 000 bootstrapping samples. For each sample we obtained by linear regression the parameters α and β (Equation 1) that minimized the root mean square error (RMSE) between experimental and predicted ΔΔG values on the whole data set or the subset of stabilizing (ΔΔG < 0.5 kcal/mol) and destabilizing (ΔΔG > 0.5 kcal/mol) mutations as previously done (1) according to the following equation:

(1) \begin{equation*} \Delta \Delta G = \alpha \Delta \Delta G_A + \beta \Delta \Delta G_B \end{equation*}

where *A* and *B* represent different prediction methods. In the case of ENCoM, ΔΔG is approximated by the calculated ΔΔS_vib_. Variance partitioning analysis (25) was performed using the ‘varpart’ function in *R* to quantify synergy between methods. Synergistic method combinations should have low shared variance and high individual variance. The results in Table 1 represent the median parameters, RMSE, error and variance of the bootstrapped ensemble.

**Table 1.** RMSE and variance for different linear combinations of methods

Methods		Parameters		Median bootstrapped RMSE			Variance		
A	B	α	β	All	Stabilizing	Destabilizing	A	B	Shared
ENCoM	FoldX	−1.12	0.38	1.24 ± 0.14	1.45 ± 0.39	1.40 ± 0.17	0.12	0.24	0.05
ENCoM	Rosetta	−1.14	0.46	1.32 ± 0.16	1.70 ± 0.41	1.43 ± 0.21	0.14	0.15	0.04
FoldX	DMutant	0.34	0.50	1.33 ± 0.15	1.42 ± 0.40	1.54 ± 0.20	0.14	0.06	0.15
FoldX	Eris	0.35	0.14	1.36 ± 0.18	1.61 ± 0.40	1.54 ± 0.26	0.16	0.01	0.12
FoldX	Rosetta	0.31	0.20	1.37 ± 0.17	1.67 ± 0.44	1.55 ± 0.23	0.10	0.00	0.18
DMutant	Rosetta	0.59	0.43	1.37 ± 0.16	1.60 ± 0.41	1.55 ± 0.24	0.10	0.07	0.11
ENCoM	Eris	−1.30	0.48	1.37 ± 0.20	1.63 ± 0.36	1.52 ± 0.35	0.16	0.12	0.01
DMutant	Eris	0.72	0.28	1.40 ± 0.19	1.48 ± 0.37	1.63 ± 0.36	0.14	0.06	0.06
Rosetta	Eris	0.41	0.15	1.43 ± 0.18	1.87 ± 0.41	1.56 ± 0.28	0.07	0.02	0.11
ENCoM	DMutant	−0.81	0.68	1.49 ± 0.17	1.40 ± 0.40	1.77 ± 0.21	0.03	0.07	0.14

Table 1 confirms our previous results showing that ENCoM has a high degree of synergy (high individual variance and low shared variance) with existing methods, that is, the combination of ENCoM to other methods is beneficial. Furthermore, combinations involving ENCoM are more beneficial than those between other methods (with the exception of the combination with DMutant). The best predictive method is a combination of ENCoM and FoldX, with the best (lowest) median RMSE values across bootstrapping samples for all mutations combined or destabilizing ones and third best median RMSE on stabilizing mutations.

Based on the results above, the final predicted ΔΔG values correspond to the linear combination of the predictions by vibrational entropy based ENCoM calculations and the enthalpy-based FoldX3.0 beta 6 (26) (Supplementary Figure S1). Our previous results (1) showed that most methods are biased toward the prediction of destabilizing mutations and are at best equal to random in predicting stabilizing mutations. The results in Table 1 extend our previous results in that it tests combinations of methods not involving ENCoM and confirm that the combination of ENCoM and FoldX is the best overall.

### Effect of mutations on dynamics

We also predict the effect of mutations on the flexibility of individual residues via the calculation of predicted b-factors using ENCoM alone (1). For each modeled mutant we provide a coordinate file and a PyMOL script to color residues as a function of the predicted effect of the mutation on the flexibility of the entire protein with respect to the wild type (Supplementary Figure S1). Residues are colored in a blue (less flexible) to red (gain of flexibility) relative to the wild type. The color range is scaled relative to the maximum absolute difference of predicted b-factor differences between wild type and all mutants across residues or three times the standard deviation of their absolute difference, whichever is smaller. Thus, the color assignment can only be used to compare different mutations that were run as part of the same job. Predicted b-factors are provided in graphical form in the web interface and numerically in a .csv file.

### Benefits of a combined analysis of stability and dynamics

The dynamic properties of individual residues are closely related to protein function. The rigidification of an enzyme may lead to a decrease in its activity (15,27,28). The increase in rigidity is often associated to an increase in thermal stability and numerous studies have analyzed the balance between protein stability and protein function (29–33). Some of the best examples come from thermophilic enzymes that display decreased activity and increased rigidity at room temperature (34). Considering that existing methods for the prediction of the effect of mutations on stability do not provide any insight into their effect on dynamics, mutations predicted with such methods need to be further analyzed or risk achieving increased stability at the expense of disrupting important aspects of dynamics. Therefore, the combined analysis of the effect of mutations on stability and dynamics with the ENCoM server may help users perform a more informed choice of mutations for protein engineering applications and understand the effect of mutations in broader terms. We provide detailed results for each mutation requested as well as a summary of the results for the top 25 stabilizing mutations with the contribution of each scoring function to the prediction of stability and the effect of mutation on the b-factors (Supplementary Figure S1). If the input includes more than two mutated residues and two types of mutations, a heatmap is generated representing the predicted ΔΔG of every position and mutant type. This heatmap may help identify hotspots affecting stability.

### Generation of conformational ensembles

Considering proteins in terms of conformational ensembles rather than single rigid structures has been shown to be beneficial in a number of different applications: small molecule docking (35–37), protein–protein docking (38,39) and protein modeling and design (40). Normal modes provide a means to generate conformational ensembles. However, care needs to be taken in the details of how the exploration of the relevant modes is performed. Existing NMA web-servers for the generation of conformational ensembles do not sample uniformly even the most accessible modes and as such provide a biased and incomplete image of the conformational landscape for a given protein structure. In particular, looking at the motions of any one mode individually does not represent the real situation where any one conformation is the linear combination of all modes. Furthermore, the models returned are the result of Cartesian translations of groups of atoms along an individual mode, thus do not respect even the most basic geometric constraints of bond angles and distances. We address these problems by performing a uniform sampling of modes selected by the user and rebuilding every conformation using Modeller with the ENCoM generated structure as a template as described below.

The conformational sampling interface works as follows. First, normal modes are calculated using the input PDB structure. Second, considering that Eigenvectors are orthonormal, an ensemble of amplitudes is found for each Eigenvector that respects the maximum RMSD distortion from the input and minimum distortion between conformations selected by the user. A new position for each atom in the protein structure is calculated from the translation of its Cartesian coordinates along the direction of movement associated to each Eigenvector by an amount defined by the selected amplitude. Thus, each combination of amplitudes for the selected normal modes generates a different structure. However, some combinations of amplitudes would effectively create a final structure with RMSD larger than the maximum chosen by the user, such combinations are removed. With the methodology described above, the ENCoM server generates an exhaustive and uniform sampling of the selected modes. Users should notice that the first six normal modes represent the translation and rotation of the entire structure and do not represent internal movements. The first mode representing internal movements is the 7^th^ mode but users may choose as starting mode any other subsequent mode.

Depending on the initial parameters, it might be impossible to have conformations that are a combination of all the modes. The number of generated structures grows combinatorially with the number of modes. To decrease computational time, the analysis is limited to at most 350 conformations. If the choice of parameters exceeds this limit, the server automatically increases the minimum RMSD between conformations in order produce at most 350 structures, thus guaranteeing a uniform sampling of conformational space within the choice of normal modes selected by the user. Lastly, every conformation generated by ENCoM is rebuilt using Modeller with the normal-mode-generated structure serving as template. This last step maps the geometrically non-realistic conformations generated by the linear combination of amplitudes for the different normal modes into the closest possible geometrically plausible structure. In addition to obtaining a structure with correct backbone angles and distances, Modeller will repackage side-chains into the new conformation. Every conformation rebuilt is provided as an individual PDB file.

In order to provide a convenient way to visualize all the generated conformations, the set of conformations is ordered in a way to minimize the RMSD between consecutive conformations to yield a smooth trajectory. It is important to note that this trajectory represents a rough morphing between structures and not a dynamics in conformational space. Mathematically, this reordering is essentially equivalent to the NP hard traveling salesman problem. However, given that this trajectory is provided only for visualization purposes, we utilize a simple heuristics to find an approximate solution. Simply, starting from every structure we generate the path that minimizes the sum RMSD values and choose that with the lowest sum. The visualization pathway is provided as a single PDB file containing consecutive states representing the ordering of individual conformations described above. We also provide a text file with the pairwise RMSD between every conformation and the amplitude applied on each normal mode employed to build each provided model. Lastly, we also produce individual PDB files with consecutive states demonstrating each individual normal mode of movement for each of the first 20 internal modes (Supplementary Figure S2).

## CONCLUSIONS

The ENCoM server offers easy access to powerful NMA-based predictions of the effect of mutations on thermostability and dynamics of single residues and generates comprehensive, geometrically realistic conformational ensembles.

## SUPPLEMENTARY DATA

Supplementary Data are available at NAR Online.

SUPPLEMENTARY DATA
