# Supplementary material for: ENCoM server: exploring protein conformational space and the effect of mutations on protein function and stability
Source: Nucleic Acids Res. 2015 Apr 16;43(Web Server issue):W395–400. doi: 10.1093/nar/gkv343 (PMC4489264; doi:10.1093/nar/gkv343)
Supplement: SUPPLEMENTARY DATA [file supp_43_W1_W395__index.html]

ENCoM server: exploring protein conformational space and the effect of mutations on protein function and stability — SUPPLEMENTARY DATA 

# ENCoM server: exploring protein conformational space and the effect of mutations on protein function and stability

## SUPPLEMENTARY DATA

**Files in this Data Supplement:**

- SUPPLEMENTARY DATA
