# Supplementary material for: ENCoM server: exploring protein conformational space and the effect of mutations on protein function and stability
Source: Nucleic Acids Res. 2015 Apr 16;43(Web Server issue):W395–400. doi: 10.1093/nar/gkv343 (PMC4489264; doi:10.1093/nar/gkv343)
Supplement: SUPPLEMENTARY DATA [file supp_gkv343_nar-00356-web-b-2015-File003.docx]

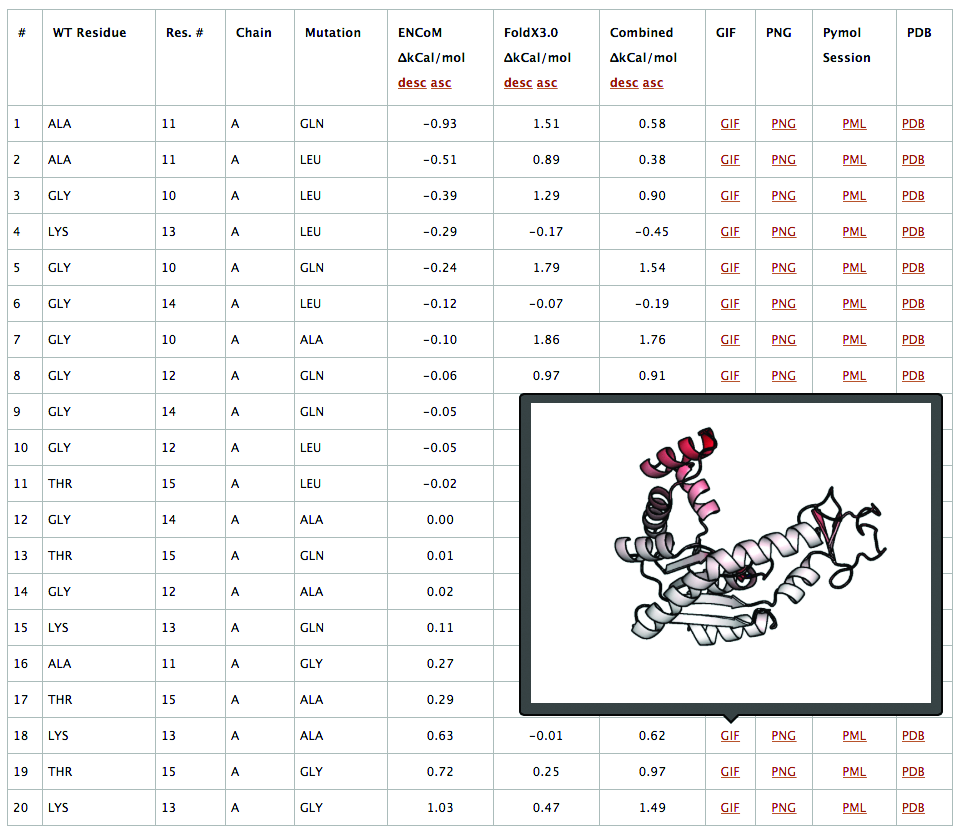


Figure S1. Results for the effect of mutations on Adenylate kinase (PDB ID 4AKE). Each amino acid from positions 10 to 15 in chain A was mutated to Q, A, L or G. The weighted predictions of ENCoM and FoldX individually or combined are shown in the respective columns are shown for every mutant. The effect of each mutation on protein flexibility can be quickly assessed graphically by hovering over the PNG or GIF links as shown. Users can download each image individually as well a PyMOL script used to generate the image and the modeled mutated structures in PDB format.


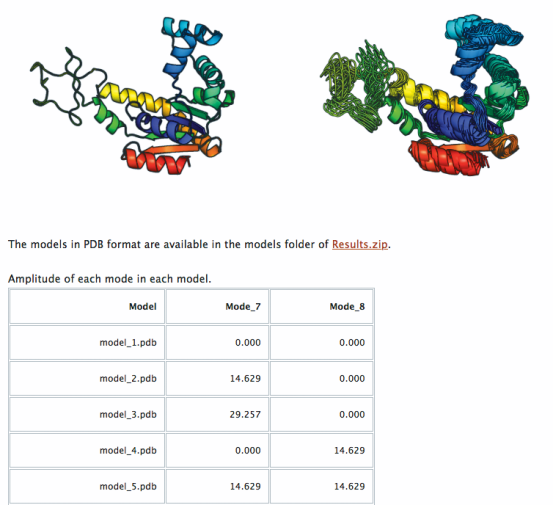


Figure S2. Screen shot of the results displayed for conformational sampling in the web interface. Adenylate kinase (4AKE) conformations were generated using the first 2 slowest non-trivial modes with a maximum RMSD distortion of 2.0 Å and a step of 1.0 Å RMSD distortion per conformation per mode. An image showing all the generated conformations and a GIF showing the reordered model trajectory is displayed. A table of the amplitudes applied to each mode on each model is shown.
